# Conference on Medical Artificial Intelligence: Analysis Should be Based on Balanced Education and Audience

**DOI:** 10.31662/jmaj.2025-0477

**Published:** 2025-12-05

**Authors:** Shigeki Matsubara

**Affiliations:** 1 Department of Obstetrics and Gynecology, Jichi Medical University, Tochigi, Japan; 2 Department of Obstetrics and Gynecology, Koga Red Cross Hospital, Koga, Japan; 3 Medical Examination Center, Ibaraki Western Medical Center, Chikusei, Japan

**Keywords:** artificial intelligence, attendee, conference, understanding

## To the Editors,

Islam et al.’s ^(1)^ study is significant. They evaluated a conference on medical artificial intelligence (AI). After the conference, attendees reported a greater understanding of medical AI, noting its benefits more clearly and expressing less concern. I have two suggestions. Although Islam et al. ^(1)^ mentioned them, some clarification may complement their data.

First, we must consider selection bias. Of 3,000 conference attendees, about 100 were estimated to have observed this session at any given time, and only 43 were analyzed. Thus, the study subjects were a highly selected fraction. Those 43, having chosen this session, were more likely to be interested in the topic, which would lead to their better understanding. With participants of different characteristics, the results might have differed.

Second, we must also consider what was taught. This conference aimed to help attendees “better understand” medical AI, yet ‘it was particularly targeted toward “encouraging” clinical professionals to use it.’ A digital innovation consultant led the hands-on session. How and to what extent medical AI should be used, especially in real-world practice, is yet to be determined ^(2), (3)^. Many seek a proper “balance” between benefits and pitfalls, which lie on a spectrum without a fixed point. I question whether the stance of the planners was “neutral,” close to the balance now considered. Although the session included “Troubles with AI,” the overall stance may still have leaned too much toward “encouraging” AI use.

I have humble propositions to reduce these possible biases. First, comparisons could be made with other sessions. Since there were many, one might examine several. The question of “increased understanding” can be asked irrespective of theme. Other sessions were also expected to consist of interested attendees: if the AI session showed a much lower score, the interpretation would differ. Second, two sub-sessions could be provided: one on the “merits” of AI and the other on its “dangers.” Comparing the data may show that different stances of planners lead to different results.

Knowing what actually happened is vital. Islam et al. ^(1)^ showed how the meeting was conducted, and describing this fact is valuable. Face-to-face discussion is also important to enhance personal understanding ^(4)^. This is especially true for AI, as the topic is new, involves multiple specialties ^(5)^, and generates more papers than any individual can digest. Islam et al.’s ^(1)^ article is useful when we organize such conferences. Irrespective of one’s stance, we must learn medical AI. Their paper encouraged us.

## Article Information

### Author Contributions

Shigeki Matsubara designed this study, and wrote, edited, and approved the final manuscript. Shigeki Matsubara meets the ICMJE criteria for authorship.

### Conflicts of Interest

None

### Ethics Approval

Jichi Medical University does not demand IRB approval for this type of study. Patient anonymity preservation and informed consent for reporting are not applicable.

### Data Availability Statement

Data sharing is not applicable to this article as no new data were created or analyzed in this study.
